# Astrocytes are involved in the formation of corpora amylacea-like structures from neuronal debris in the CA1 region of the rat hippocampus after ischemia

**DOI:** 10.3389/fncel.2023.1308247

**Published:** 2023-12-22

**Authors:** Tae-Ryong Riew, Ji-Won Hwang, Xuyan Jin, Hong Lim Kim, Sharon Jiyoon Jung, Mun-Yong Lee

**Affiliations:** ^1^Department of Anatomy, Catholic Neuroscience Institute, College of Medicine, The Catholic University of Korea, Seoul, Republic of Korea; ^2^Department of Biomedicine and Health Sciences, College of Medicine, The Catholic University of Korea, Seoul, Republic of Korea; ^3^Integrative Research Support Center, Laboratory of Electron Microscope, College of Medicine, The Catholic University of Korea, Seoul, Republic of Korea; ^4^Technological Convergence Center, Korea Institute of Science and Technology (KIST), Seoul, Republic of Korea

**Keywords:** osteopontin, astrocyte, microglia, corpora amylacea, global ischemia, calcification, correlative light and electron microscopy

## Abstract

Recently, we demonstrated that the corpora amylacea (CA), a glycoprotein-rich aggregate frequently found in aged brains, accumulates in the ischemic hippocampus and that osteopontin (OPN) mediates the entire process of CA formation. Therefore, this study aimed to elucidate the mechanisms by which astrocytes and microglia participate in CA formation during the late phase (4–12 weeks) of brain ischemia. Based on various morphological analyses, including immunohistochemistry, *in situ* hybridization, immunoelectron microscopy, and correlative light and electron microscopy, we propose that astrocytes are the primary cells responsible for CA formation after ischemia. During the subacute phase after ischemia, astrocytes, rather than microglia, express *Opn* messenger ribonucleic acid and OPN protein, a surrogate marker and key component of CA. Furthermore, the specific localization of OPN in the Golgi complex suggests that it is synthesized and secreted by astrocytes. Astrocytes were in close proximity to type I OPN deposits, which accumulated in the mitochondria of degenerating neurons before fully forming the CA (type III OPN deposits). Throughout CA formation, astrocytes remained closely attached to OPN deposits, with their processes exhibiting well-developed gap junctions. Astrocytic cytoplasmic protein S100β, a calcium-binding protein, was detected within the fully formed CA. Additionally, ultrastructural analysis revealed direct contact between astroglial fibrils and the forming facets of the CA. Overall, we demonstrated that astrocytes play a central role in mediating CA formation from the initial stages of OPN deposit accumulation to the evolution of fully formed CA following transient ischemia in the hippocampus.

## 1 Introduction

Osteopontin (OPN) is a secreted phosphoprotein involved in various pathophysiological processes (O'Regan and Berman, 2000; Denhardt et al., 2001; Zhu et al., 2017; Rosmus et al., 2022) and regulates ectopic brain calcification (Maetzler et al., 2010; Shin et al., 2011, 2012; Riew et al., 2017; Grand Moursel et al., 2019). We recently reported that OPN is engaged in the conversion of degenerated neuronal elements into corpora amylacea (CA)-like structures during the prolonged phase after an initial ischemic episode (Riew et al., 2022). Our study comprehensively delineates the entire process of CA formation triggered by ischemia, clarifying the origin and development of CA during neurodegeneration. As CA acts as a container for waste substances and participates in brain clearance mechanisms (Augé et al., 2017, 2019; Navarro et al., 2018; Riba et al., 2019, 2021), our study suggests a novel role for OPN in removing deleterious neuronal debris via the formation of CA-like structures.

Information regarding the formation and function of CA in the brain is substantial; however, its cellular origin and underlying mechanisms remain debated. Several studies, including ours, have revealed that the cellular origin of CA is neuronal debris (Selmaj et al., 2008; Wilhelmus et al., 2011; Doehner et al., 2012; Notter and Knuesel, 2013; Riew et al., 2022); however, others suggest that CA is derived from astrocytes because of its location within astrocytic processes and its close relationship with astrocytes (Ramsey, 1965; Palmucci et al., 1982; Sbarbati et al., 1996; Leel-Ossy, 2001; Manich et al., 2016; Navarro et al., 2018; Augé et al., 2019; Wander et al., 2022). In this context, our recent findings demonstrated that astrocytes are closely related to degenerating neuronal debris during the conversion of degenerated neuronal debris into CA, even though they are not the cellular origin of CA (Riew et al., 2022). This finding implies that astrocytes play essential roles in CA formation. Most studies examining the association between CA and neuroglial cells have focused on astrocytes. However, some studies have reported that microglia or macrophages can also interact with and phagocytose CA (Riba et al., 2019; Wander et al., 2022). Despite extensive research aimed at understanding the cellular mechanisms behind CA formation, the functional roles of these two glial cells, astrocytes and microglia, in CA formation remain to be fully elucidated.

Therefore, this study aimed to elucidate the mechanisms by which astrocytes and microglia participate in CA formation during the chronic phase (4–12 weeks) following brain ischemia. We conducted confocal microscopy and ultrastructural analysis utilizing conventional transmission electron microscopy (TEM) and correlative light and electron microscopy (CLEM). Our results provide new information that astrocytes are actively involved in the transformation of degenerated neuronal elements into CA-like structures during the late phase after an initial ischemic episode.

## 2 Materials and methods

### 2.1 Animals and surgery

All surgical interventions and presurgical and postsurgical animal care were performed in accordance with the Laboratory Animals Welfare Act, Guide for the Care and Use of Laboratory Animals, and Guidelines and Policies for Rodent Survival Surgery provided by the Institutional Animal Care and Use Committee (IACUC) at the College of Medicine of The Catholic University of Korea (Approval No.: CUMS-2020-0041-03). The IACUC and Department of Laboratory Animals at the Catholic University of Korea, Songeui Campus, accredited the Korea Excellence Animal Laboratory Facility of the Korea Food and Drug Administration in 2017 and reaccredited it in 2021. Additionally, full accreditation was acquired from the Association for Assessment and Accreditation of Laboratory Animal Care International in 2018 and was reaccredited in 2022. All efforts were made to minimize animal suffering and reduce the number of animals used.

Adult male Sprague-Dawley rats weighing 250–300 g (Orient Bio, Seongnam, Republic of Korea) were used in this study. These animals were housed in groups of two to three per cage under controlled environmental conditions, maintaining a constant temperature (22 ± 5°C) and humidity (50 ± 10%), with food (gamma ray-sterilized diet) and water (autoclaved tap water) available *ad libitum*. Global transient forebrain ischemia was induced using a modified version of the four-vessel occlusion and reperfusion method described by Pulsinelli and Brierley (1979) and Lee et al. (2002). This procedure involved the electrocauterization and cutting of the vertebral arteries to halt circulation in these vessels and occlusion of common carotid arteries for 10 min using miniature aneurysmal clips after 24 h. Body temperatures (measured rectally) were maintained at 37.5 ± 0.3°C with a heating lamp during and after ischemia. Sham-operated rats with cauterized vertebral arteries and ligatures placed around the carotid arteries were used as controls. Animals demonstrating the absence of righting reflex post-ischemia were categorized as having ischemia, and prominent neuronal loss in the CA1 region was confirmed using hematoxylin-eosin-stained sections. None of the animals exhibited seizures or mortality following reperfusion or sham surgery. Postoperative care and monitoring were performed twice a day for 1 week to assess behavioral changes and general conditions, including body weight and temperature. The overall mortality rate among ischemic rats was < 20%, and all sham-operated rats survived postoperatively.

Animals were sacrificed 2-, 4-, 8-, 12-, or 24-weeks post- reperfusion (*n* = 3–8 rats per time point for the ischemic group and *n* = 3 rats per time point for the sham-operation group). After anesthesia with zolazepam (20 mg/kg i.p.) and xylazine (7.5 mg/kg i.p.), the animals underwent transcardial perfusion with 4% paraformaldehyde in 0.1 M phosphate buffer (pH 7.4). Subsequently, the brain tissues were cryoprotected and frozen for light microscopy.

### 2.2 *In situ* hybridization and immunofluorescence analysis

Three animals from sham-operated group and three animals each from 2- and 4-weeks post-reperfusion groups were utilized for *in situ* hybridization. Antisense and sense riboprobes for *Opn* (GenBank accession number: M14656, nucleotides 426-991) were used. Coronal cryostat sections (25 μm thick) were hybridized with antisense or sense riboprobes labeled with digoxigenin as previously described (Shin et al., 2011; Riew et al., 2019). Additionally, three animals from sham-operated group, eight animals each from 4, 8, or 12 weeks post-reperfusion group, and three animals from 24 weeks post-reperfusion group were used for fluorescence immunohistochemistry, free-floating sections (25 μm thick) were blocked with blocking solution (a mixture of 10% normal serum, 1% bovine serum albumin, and 0.1% triton X-100 or a mixture of 0.2% gelatin, 1% bovine serum albumin, and 0.05% saponin). Subsequently, they were incubated at 4°C overnight with a mixture of primary antibodies summarized in Table 1. The secondary antibodies were Cy3-conjugated donkey anti-goat/mouse antibody (1:2,000; Jackson ImmunoResearch, West Grove, PA, USA), Cy3-conjugated streptavidin (1:2,000; Jackson ImmunoResearch), Alexa 488 donkey anti-rabbit/mouse antibody (1:300; Thermo Fisher, Waltham, MA, USA), and Alexa 647 donkey anti-chicken/goat antibody (1:300; Thermo Fisher). Negative controls for immunofluorescence staining involved omitting primary or secondary antibodies. Cell nuclei were counterstained using 4,6-diamidino-2-phenylindole (DAPI, 1:2000; Roche, Mannheim, Germany) for 10 min. The slides were viewed under a confocal microscope (LSM 900 with Airyscan; Carl Zeiss Co. Ltd., Oberkochen, Germany), equipped with four lasers (Diode 405, Argon 488, HeNe 543, and HeNe 633), maintained under constant viewing conditions. Structured illumination microscopy (SIM) imaging was performed using an Elyria 7 (Carl Zeiss) equipped with 405, 488, 561, and 642 nm laser lines at a 63x/NA1.4 objective. The raw images comprised 13 phase images per plane per channel and were acquired with a Z-distance of 0.200 μm, with an exposure time of 50 ms for all images. The image size was 2048 × 2048 pixels, and SIM reconstruction was performed using the Zen software with automatic settings. Images were converted to TIFF format, and contrast levels were adjusted using Adobe Photoshop v.13.0.

**Table 1:** Details of primary antibodies used.

**Antigen**	**Marker**	**Dilution**	**Manufacturing details**	**Host**
OPN	Corpora amylacea	1:1,000	R&D systems, Minneapolis, MN, USA, AF808	Goat
		1:300	American Research Products, Waltham, MA, USA, 01-20002	Mouse
S100β	Astrocyte	1:500	Abcam, Cambridge, UK, ab52642	Rabbit
S100a10	Astrocyte	1:100	Abcam, Cambridge, UK, ab50737	Chicken
Glial fibrillary acidic protein (GFAP)	Astrocyte	1:500	Millipore, Burlington, MA, USA, AB5541	Chicken
		1:700	Millipore, Burlington, MA, USA, MAB360	Mouse
Ionized calcium-binding adaptor molecule 1 (Iba1)	Microglia, macrophage	1:500	Wako Pure Chemical Co., Osaka, Japan, 019-19741	Rabbit
		1:400	Abcam, Cambridge, UK, ab5076	Goat
Lysosomal-associated membrane protein 1 (LAMP1)	Lysosome	1:100	Abcam, Cambridge, UK, ab24170	Rabbit
Golgi matrix protein 130 kDA (GM130)	Golgi complex	1:150	BD Transduction Laboratories, San Jose, Ca, USA, 610822	Mouse
78-kDa glucose-regulated protein (GRP78)	Endoplasmic reticulum	1:2000	Abcam, Cambridge, UK, ab21685	Rabbit
Connexin-43 (Cx-43)	Gap junction	1:300	Sigma-Aldrich, St. Louis, MO, USA, C6219	Rabbit

### 2.3 Quantitative analysis and statistics

For quantitative analysis, confocal images were captured using a 63 × objective lens with an Airyscan under constant viewing settings. To compare glial contacts of OPN deposits in the hippocampal CA1 region, we used 78.01 × 78.01 μm images of 6.8 μm Z stacks, with 0.3 μm optical sections, from eight different animals. The perimeter of each OPN deposit and Iba1 or GFAP-labeled perimeter were measured using Zen 3.0 blue (Carl Zeiss).

Statistical significance was determined through the Student's *t-*test, with considering differences as significant when *P* values were < 0.05. The number of animals assessed, and the *P* values are indicated in figure legends and the graphs. All statistical analyses were conducted using the Prism 7 software (GraphPad Software Inc., San Diego, CA, USA).

### 2.4 Electron microscopic analysis

For pre-embedding immunoelectron microscopy, floating vibratome sections (50 μm thick) from control (*n* = 3) and ischemic rats at 2-, 4-, 8-, and 12-weeks post-reperfusion (*n* = 3 rats/time point) were used. Immunohistochemical staining was performed with goat anti-OPN (R&D Systems, 1:1,000) and visualized using peroxidase-labeled donkey anti-goat Immunoglobulin G (Jackson ImmunoResearch, 1:150) and 0.05% 3,3-diaminobenzidine tetrahydrochloride (DAB) as a chromogen. The tissues were post-fixated with 2.5% glutaraldehyde and 1% osmium tetroxide (OsO4) and embedded in epon 812 (Polysciences, Warrington, PA, USA). Ultrathin sections of 70–90 nm thicknesses were prepared and observed under an electron microscope (JEM 1010; JEOL, Tokyo, Japan) with slight uranyl acetate staining.

For conventional TEM, vibratome sections (50 μm thick) from control (*n* = 3) and ischemic rats at 2, 4, 8, and 12 weeks after reperfusion (*n* = 3 rats/time point) were used. Trimmed tissues were post-fixed with 2.5% glutaraldehyde and 1% OsO4 and then processed further for epon812 embedding.

For the CLEM study, semi-thin cryosections (2 μm thick) of cryoprotected hippocampus from control (*n* = 3) and ischemic rats at 2, 4, 8, and 12 weeks after reperfusion (*n* = 3 rats/time point) were cut at −100°C with a glass knife in a Leica EM UC7 ultramicrotome equipped with an FC7 cryochamber (Leica, Wetzlar, Germany). The sections were immuno-labeled with goat anti-OPN (R&D Systems, 1:1,000), rabbit anti-Iba1 (Wako, 1:500), goat anti-Iba1 (Abcam, 1:400), chicken anti-GFAP (Millipore, 1:500), or rabbit anti-S100β (Abcam, 1:500). Antibody staining was visualized using Cy3-conjugated donkey anti-goat antibody (1:2,000; Jackson ImmunoResearch), Alexa 488 donkey anti-rabbit antibody (1:300; Thermo Fisher Scientific, Waltham, MA, USA), and Alexa 647 donkey anti-chicken antibody (1:300; Thermo Fisher Scientific). Sections were labeled with DAPI for 10 min. Immuno-labeled sections were photographed using confocal microscopy and processed for electron microscopy as previously described (Riew et al., 2019, 2022; Kim et al., 2021).

## 3 Results

### 3.1 Relationships between OPN deposits, astrocytes, and microglia in the pyramidal cell layer of the CA1 hippocampus after ischemia

**Figure 1:**
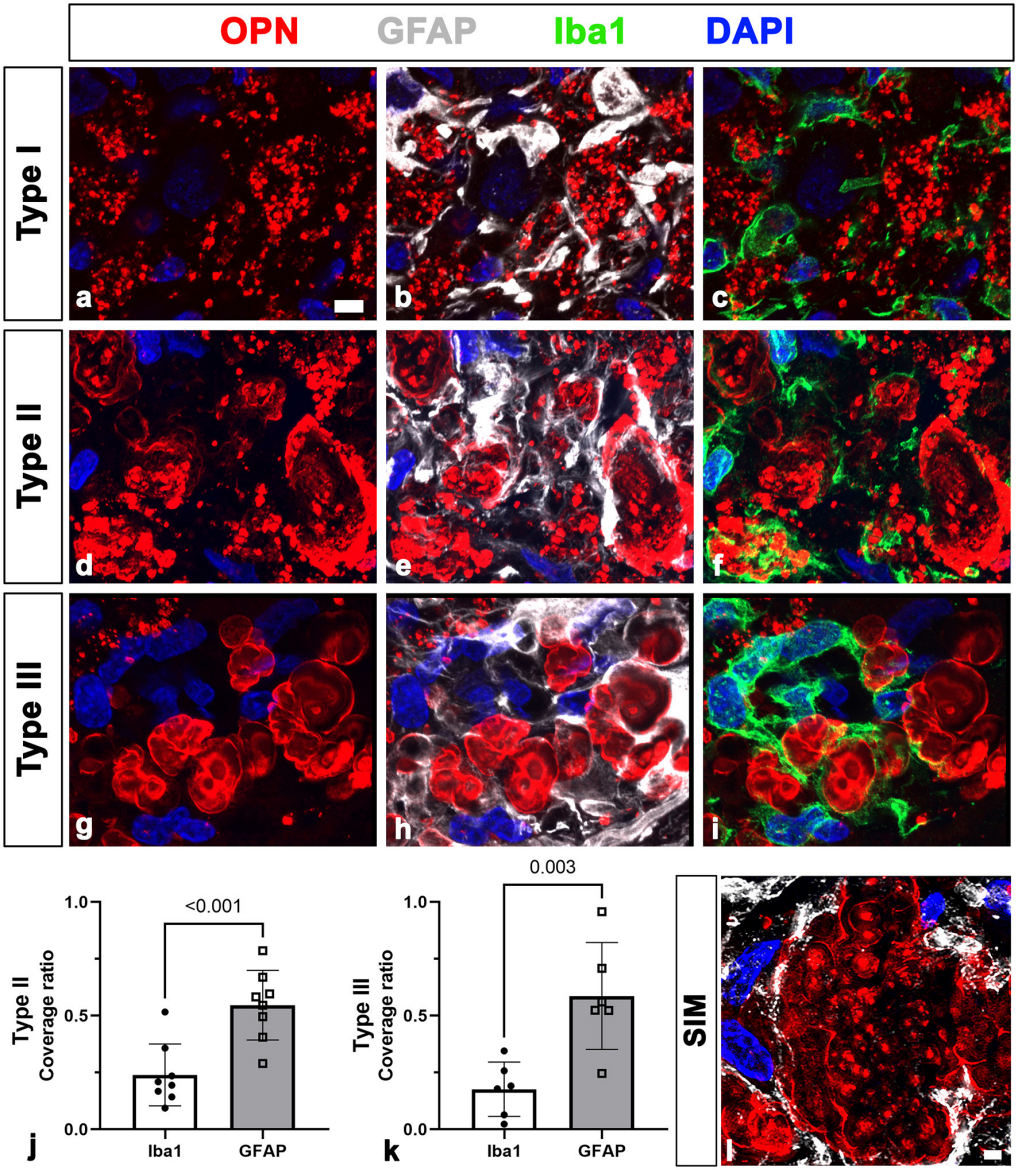
Representative images showing the spatial relationships between three types of osteopontin (OPN)-positive deposits and neuroglial cells in the pyramidal cell layer of the ischemic CA1 hippocampus. **(a–i)** Triple labeling for OPN, glial fibrillary acidic protein (GFAP), and ionized calcium-binding adaptor molecule 1 (Iba1), showing that three distinct types of OPN deposits were closely associated with astroglial processes and microglia. Subsequently, we quantified the relative proportions of OPN deposits contacted by astrocytes or microglia. **(j, k)** Quantitative analysis of the relative proportions of type II **(j)** and type III **(k)** OPN deposits contacted by astrocytes and microglia, respectively. Both types of OPN deposits were more covered by astrocytes compared with microglia, with type II OPN deposits showing significant coverage by astrocyte profiles rather than microglia (*n* = 3–5 sections from 8 rats per time point, Student's *t-*test). The data are expressed as mean ± SEM, and the numbers on each bar graph indicate the *p* values. **(l)** Structured illumination microscopy image clearly revealed that astrocytic processes almost completely and closely surrounded type III OPN deposits. Scale bars = 5 μm for **(a–i)**, 2 μm for **(l)**.

First, we assessed the spatial relationships between OPN deposits and neuroglial cells in the pyramidal cell layer of the CA1 hippocampus of ischemic rats using triple labeling with OPN and astrocyte and microglia markers, GFAP and Iba1. Consistent with our previous data (Riew et al., 2022), our findings demonstrated that the three distinct types of OPN deposits could be distinguished based on their morphological features. Type I OPN deposits exhibited granular puncta confined to the cell (Figure 1a). Type II OPN deposits featured structures of highly variable size and shape (Figure 1d). Type III OPN deposits manifested smooth-contoured, concentric layered structures with intense OPN expression detected in the peripheral and core regions (Figure 1g). These OPN deposits were closely associated with or enwrapped via astroglia processes (Figures 1b, e, h). Additionally, microglia frequently exhibited proximity to OPN deposits (Figures 1c, f, i). Subsequently, we quantified the relative proportions of OPN deposits in contact with astrocytes and microglia. Figures 1j, k show that types II and III deposits exhibited stronger associations with or are surrounded by astrocytes than by microglia, indicating that OPN deposits were closely associated with astrocytes and, to a lesser extent, microglia. SIM super-resolution microscopy clearly revealed that astrocytic processes almost surrounded type III OPN deposits (Figure 1l).

### 3.2 OPN production and secretion by reactive astrocytes in the CA1 pyramidal cell layer during the chronic phase after ischemia

**Figure 2:**
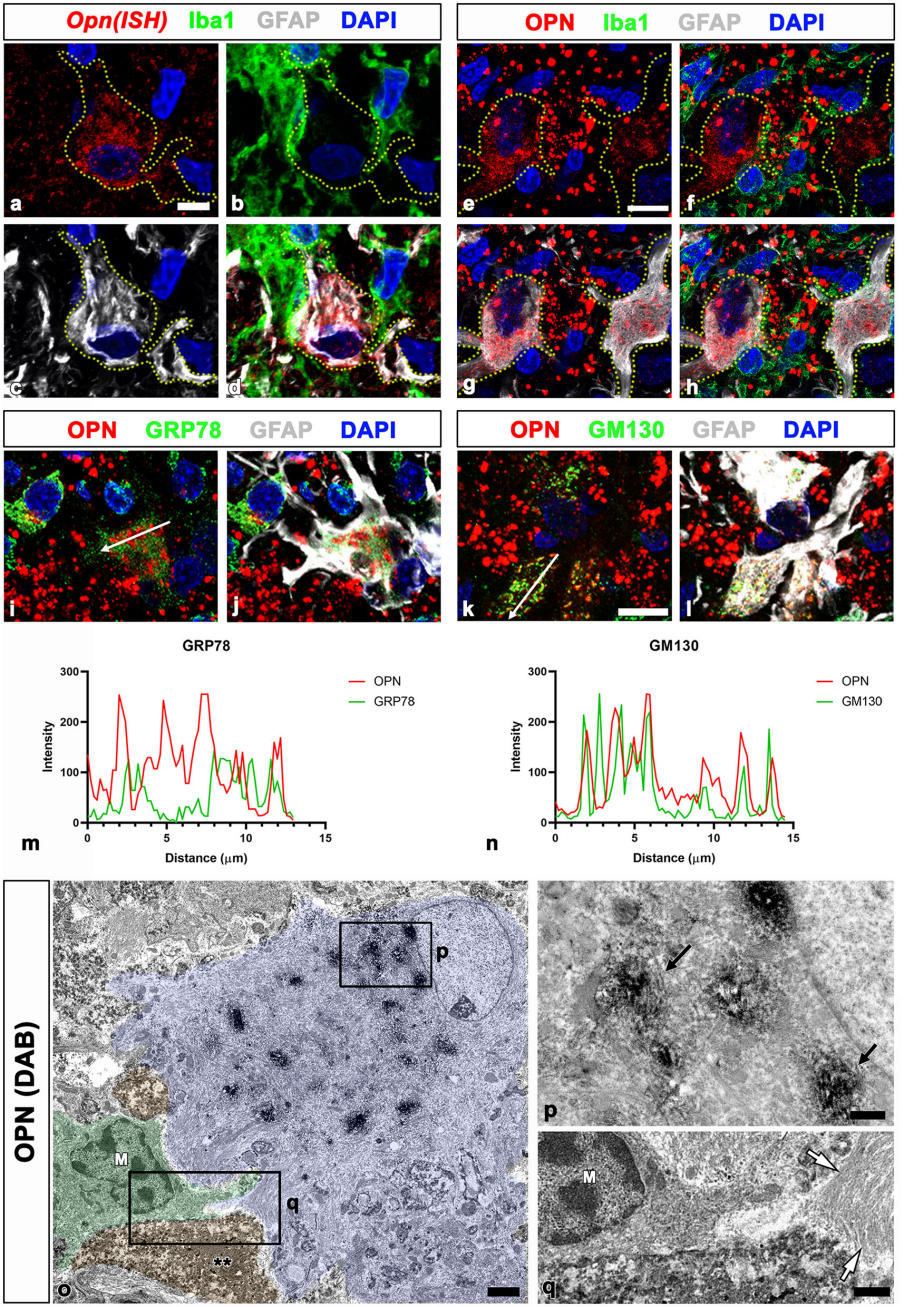
Spatial relationships between *Opn* messenger ribonucleic acid (mRNA) or protein and neuroglial cells in the pyramidal cell layer of the ischemic CA1 hippocampus. **(a–d)** Triple labeling for *Opn* mRNA, Iba1, and GFAP show that *Opn* mRNA was expressed in reactive astrocytes (yellow dotted lines outline cell boundaries), with weak or negligible expression in activated microglial cells. **(e–h)** Triple labeling for OPN, Iba1, and GFAP show that OPN protein, visible as small granular puncta (blue arrowheads), was localized to the perinuclear region of reactive astrocytes outlined by yellow dotted lines, but not within microglia. OPN deposits of various sizes and shapes, corresponding to type I OPN deposits, were observed among reactive astrocytes. **(i–l)** Triple labeling for OPN, GFAP, and GRP78 (endoplasmic reticulum protein) **(i, j)** or the cis-Golgi marker Golgi matrix protein 130 kDa (GM130) **(k, l)** show different spatial distributions of OPN and GRP78 within reactive astrocytes, whereas OPN and GM130 shared an overlapping distribution within astrocytes. **(m, n)** Histograms of the intensity profiles of OPN-positive signals and 78-kDa glucose-regulated protein (GRP78)-positive or GM130-positive signals in the indicated area [white arrows in **(i, k)**]. OPN shared overlapping spatial profiles within astrocytes with GM130 but not with GRP78. **(o–q)** Pre-embedding electron microscopy images immunostained with OPN show that OPN immunoreactivity is associated with the Golgi complex, but not with other cytoplasmic organelles or cell nuclei within reactive astrocytes. Astrocytes showing extensive hypertrophy were frequently observed in proximity to microglia (M) and shrunken dark cells (**) with homogeneous nuclei and unidentifiable organelles. The boxed areas in **(o)** are enlarged in **(p)** and **(q)**, respectively. Electron-dense 3,3-diaminobenzidine tetrahydrochloride grains for OPN were specifically localized to the saccules and tubules in the Golgi complex [arrows in **(P)**]. White arrows in **(q)** denote bundles of glial filaments in astrocytic cytoplasm. Scale bars = 5 μm for **(a–d)**, 10 μm for **(e–l)**, 2 μm for **(o)**, 0.5 μm for **(p)**, 1 μm for **(q)**.

We previously proposed that the two stages of OPN production and secretion occur in a time- and cell-dependent manner in ischemic hippocampi (Choi et al., 2007). Activated microglia produce and secrete OPN during the acute phase (< 1 week after reperfusion). In contrast, in the chronic phase (>2 weeks after reperfusion), reactive astrocytes in the CA1 pyramidal cell layer exhibited robust and sustained *Opn* messenger ribonucleic acid (mRNA) expression. Thus, we determined whether OPN, which is involved in the formation of CA-like structures in the CA1 hippocampus after ischemia, was synthesized and secreted by reactive astrocytes through triple labeling using *in situ* hybridization and immunofluorescence. In the CA1 pyramidal cell layer reperfused for 4 weeks, *Opn* mRNA was expressed in GFAP-positive reactive astrocytes. However, the expression was weak or negligible in activated microglial cells (Figures 2a–d). Thus, *Opn* mRNA expression in the chronic phase after ischemia was induced in reactive astrocytes, which was consistent with our previous data (Choi et al., 2007). OPN in reactive astrocytes was substantiated through triple labeling for OPN, GFAP, and Iba1. As shown in Figures 2e–h, OPN, visible as small granular puncta, was localized to the perinuclear region of reactive astrocytes, but was absent within microglia. Additionally, OPN deposits of various sizes and shapes observed among reactive astrocytes corresponded to type I OPN deposits, as previously reported (Riew et al., 2022).

To determine the subcellular localization of OPN in reactive astrocytes, we performed triple labeling for OPN, GFAP, and GRP78, the endoplasmic reticulum protein, or the cis-Golgi marker GM130 (Nakamura et al., 1995; Marra et al., 2001). OPN and GRP78 revealed different spatial distributions within reactive astrocytes (Figures 2i, j), whereas OPN and GM130 exhibited overlapping distributions within astrocytes (Figures 2k, l). These spatial correlations for OPN and the two cell organelle markers within reactive astrocytes were verified through the fluorescence intensity profiles of these signals (Figures 2m, n).

We used pre-embedding immunoelectron microscopy to establish the precise subcellular localization of OPN in reactive astrocytes. OPN immunoreactivity in astrocytes, characterized by highly electron-dense DAB grains, was associated with the Golgi complex, excluding other cytoplasmic organelles such as lysosomes and cell nuclei (Figures 2o, p). These astrocytes were closely associated with shrunken dark cells, exhibiting small spots of dispersed condensed chromatin within homogeneous nuclei and unidentifiable organelles (Figures 2o, q), corresponding to degenerative neurons identified in our previous work (Riew et al., 2022). These findings indicate that OPN is synthesized and secreted by reactive astrocytes but not by activated microglia in the CA1 pyramidal cell layer during the chronic phase after reperfusion.

### 3.3 Ultrastructural relationships among OPN deposits, astrocytes, and microglia within the pyramidal cell layer of the CA1 hippocampus after ischemia

**Figure 3:**
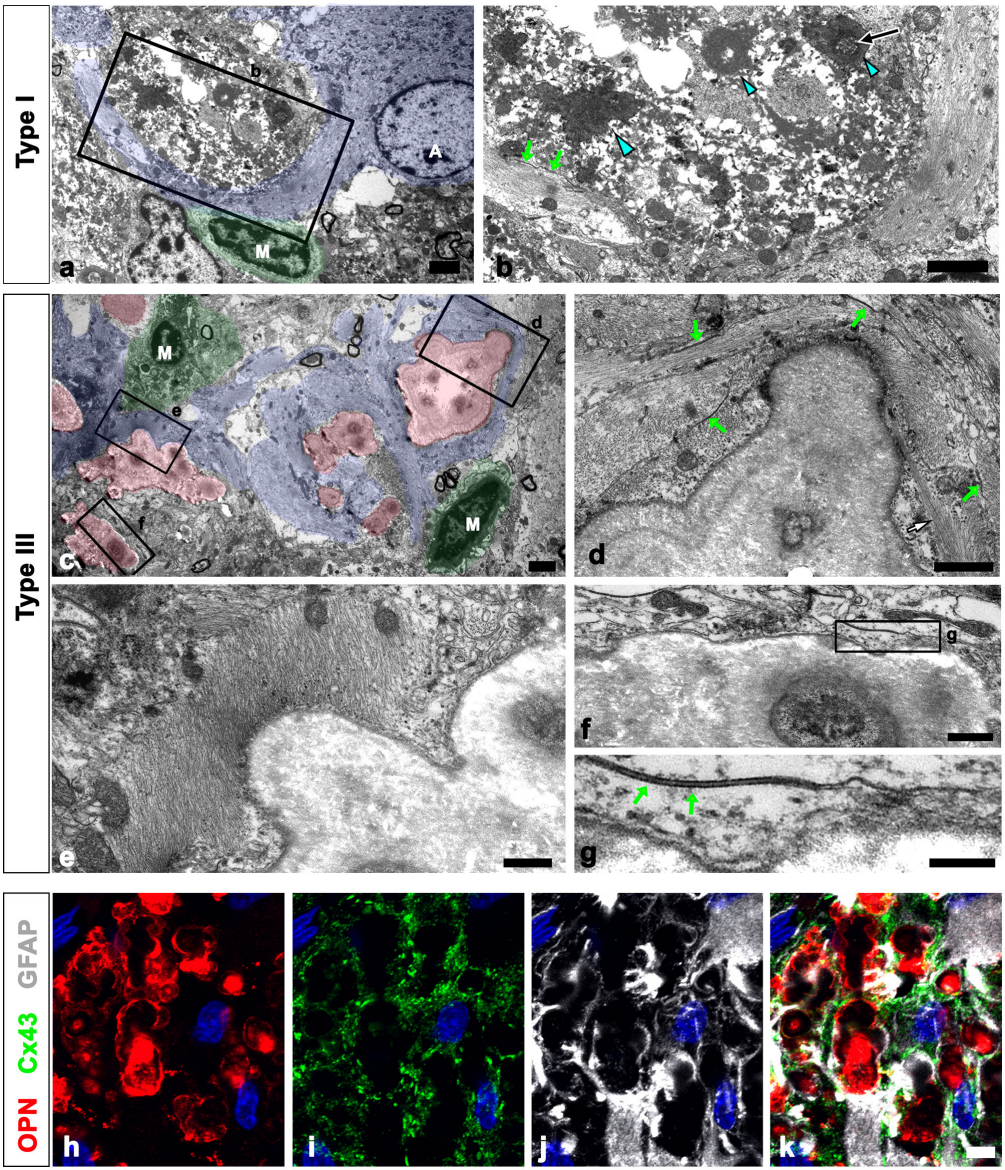
Ultrastructural relationships between OPN deposits and neuroglial cells in the pyramidal cell layer of the ischemic CA1 hippocampus. **(a, b)** Lower **(a)** and higher **(b)** magnified conventional electron microscopic images show that type I OPN deposits with electron-dense profiles [blue arrowheads in **(b)**] that contained degenerating mitochondria [arrow in **(b)**] were surrounded by direct contact with astrocytes (shaded in blue). Astrocytes [A in **(a)**] contained dense bundles of glial filaments in which gap junctions [green arrows in **(b)**] were observed. Microglia [M in **(a)**] were not in direct contact with the deposits via astroglia processes. **(c)** Conventional electron microscopic image shows that type III OPN deposits (shaded in red) with compacted and laminated structures were surrounded by astroglia processes (shaded in blue). Microglia (M, shaded in green) without obvious morphological changes of activation were observed around the OPN deposits without direct contact. **(d–g)** The boxed areas in **(c)** were enlarged in **(d–f)**, and the boxed area in **(f)** was enlarged in **(g)**. Astroglia processes encompassing type III OPN deposits comprised multiple layers with alternate concentrically laminated structures containing parallel glial fibers within each layer. These layers are connected by distinct gap junctions [green arrows in **(d, g)**] located between each layer. **(h–k)** Triple labeling for OPN, GFAP, and the primary astrocytic gap junction protein connexin43 (Cx43) show that distinct Cx43 expression was observed in astroglia processes that almost surrounded the OPN deposits. Scale bars = 2 μm for **(a–c)**, 1 μm for **(d)**, 0.5 μm for **(e, f)**, 0.2 μm for **(g)**, and 5 μm for **(h–k)**.

As shown in Figure 1, all types of OPN deposits were closely associated with astrocytes, and this pattern became more distinct from types I to III, with type III deposits being almost completely surrounded by astrocytic processes. We further examined the relationship between OPN deposits and neuroglial cells using conventional TEM. Type I OPN deposits with electron-dense profiles contained degenerating mitochondria that were surrounded by astroglia processes containing dense bundles of intermediate filaments in which gap junctions were observed (Figures 3a, b). Microglia were observed adjacent to type I OPN deposits, but not in direct contact with the deposits, as astroglia processes separated them.

Type III OPN deposits with a CA-like ultrastructural morphology, that is compacted and laminated structures, composed of tightly aggregated fibrils (Riew et al., 2022), were also surrounded by astroglia processes (Figure 3c). Astroglia processes encompassing type III OPN deposits had multiple layers with alternating concentric laminated structures containing parallel glial fibers within each layer, which were interconnected by distinct gap junctions (Figures 3d–g). Microglia showing no obvious morphological changes of activation were observed around the OPN deposits but were not in direct contact with them (Figure 3a). The presence of gap junctions within the astroglia processes surrounding the OPN deposits was substantiated through triple labeling for OPN, GFAP, and the primary astrocytic gap junction protein Cx43. The OPN deposits were almost surrounded by astroglia processes, with distinct Cx43 expression confined to these processes (Figures 3h–k).

**Figure 4:**
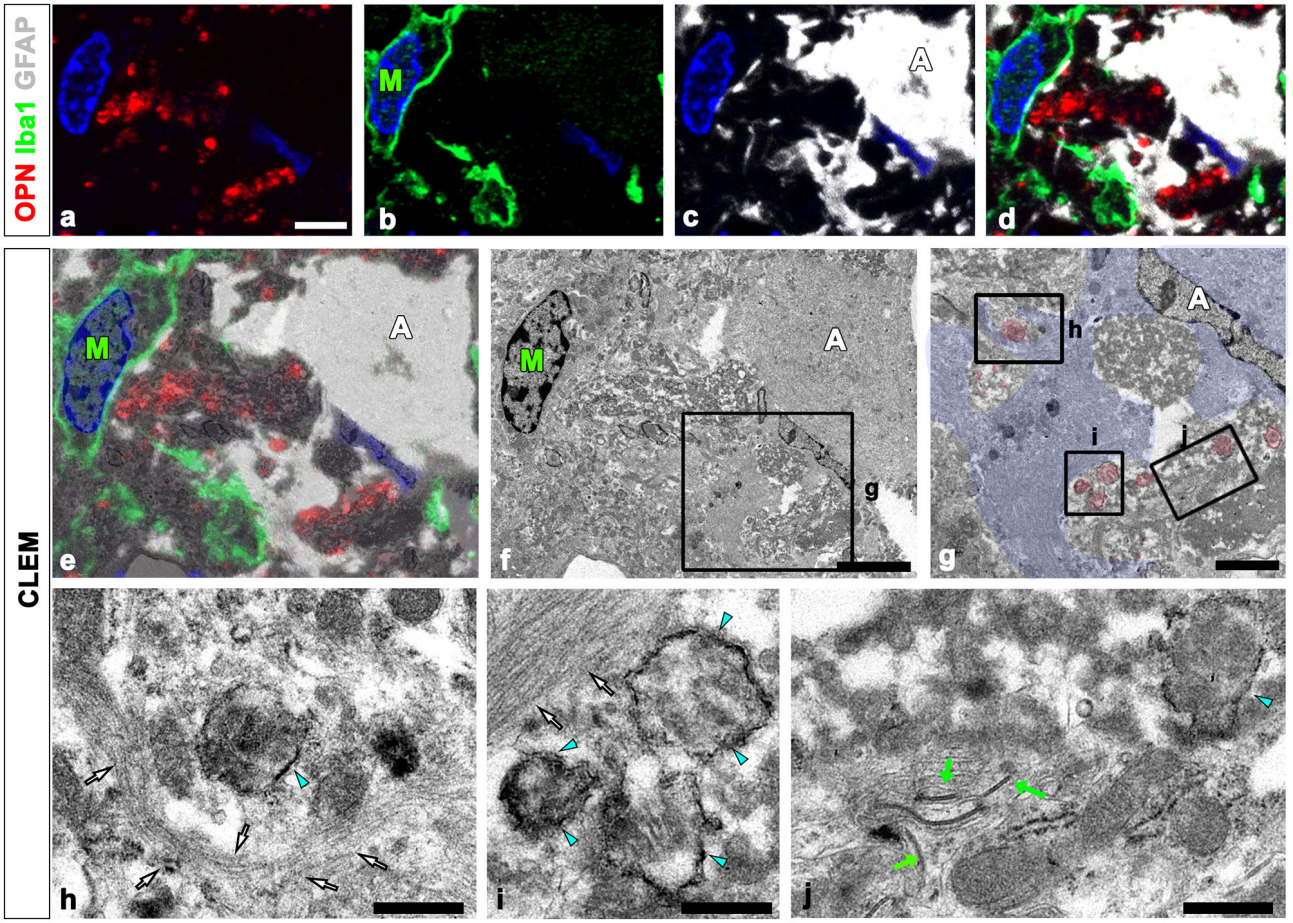
Ultrastructural relationships between OPN deposits and neuroglial cells using correlative light and electron microscopy (CLEM). **(a–f)** Confocal microscope images of a semi-thin section triple-labeled for OPN, Iba1, and GFAP **(a–d)**, the overlay image of the confocal microscope data and the corresponding electron microscopic image **(e)**, and the corresponding electron microscope image **(f)**. **(g–i)** The boxed area in **(f)** was enlarged in **(g)**, and the boxed areas in **(g)** were enlarged in **(h–j)**. OPN staining within dark degenerated cells was localized to calcified mitochondria [blue arrowheads in **(h–j)**] with electron-dense outlines corresponding to type I OPN deposits. The degenerated cells containing type I OPN deposits established direct contact with dense astroglial bundles [white arrows in **(h, i)**] of astrocytes (A; shaded in blue) where gap junctions [green arrows in **(j)**] were frequently observed. Microglia (M) were observed adjacent to the degenerated cell. Scale bars = 5 μm for **(a–f)**, 2 μm for **(g)**, 0.5 μm for **(h–j)**.

We defined the relationship between OPN deposits and neuroglial cells more precisely using CLEM. Semi-thin sections triple-labeled with OPN, GFAP, and Iba1 were initially observed using confocal microscopy (Figures 4a–d). Subsequently, these same semi-thin sections were subjected to electron microscopy. An overlay of confocal microscopy and TEM data revealed OPN staining within dark-degenerated cells and corresponded to calcified mitochondria with an electron-dense outline, which is a type I OPN deposit (Figures 4e–j), as previously reported (Riew et al., 2022). Dense bundles of parallel-running astroglia filaments surrounded these degenerated cells containing type I OPN deposits in direct contact (Figures 4h–j), with gap junctions frequently observed between these bundles (Figure 4j). However, the degenerated cells were not in direct contact with adjacent microglia (Figures 4e, f).

### 3.4 Comparison of GFAP and calcium-binding protein S100β and S100a10 in OPN deposits and astrocytes in the pyramidal cell layer of the CA1 hippocampus after ischemia

**Figure 5:**
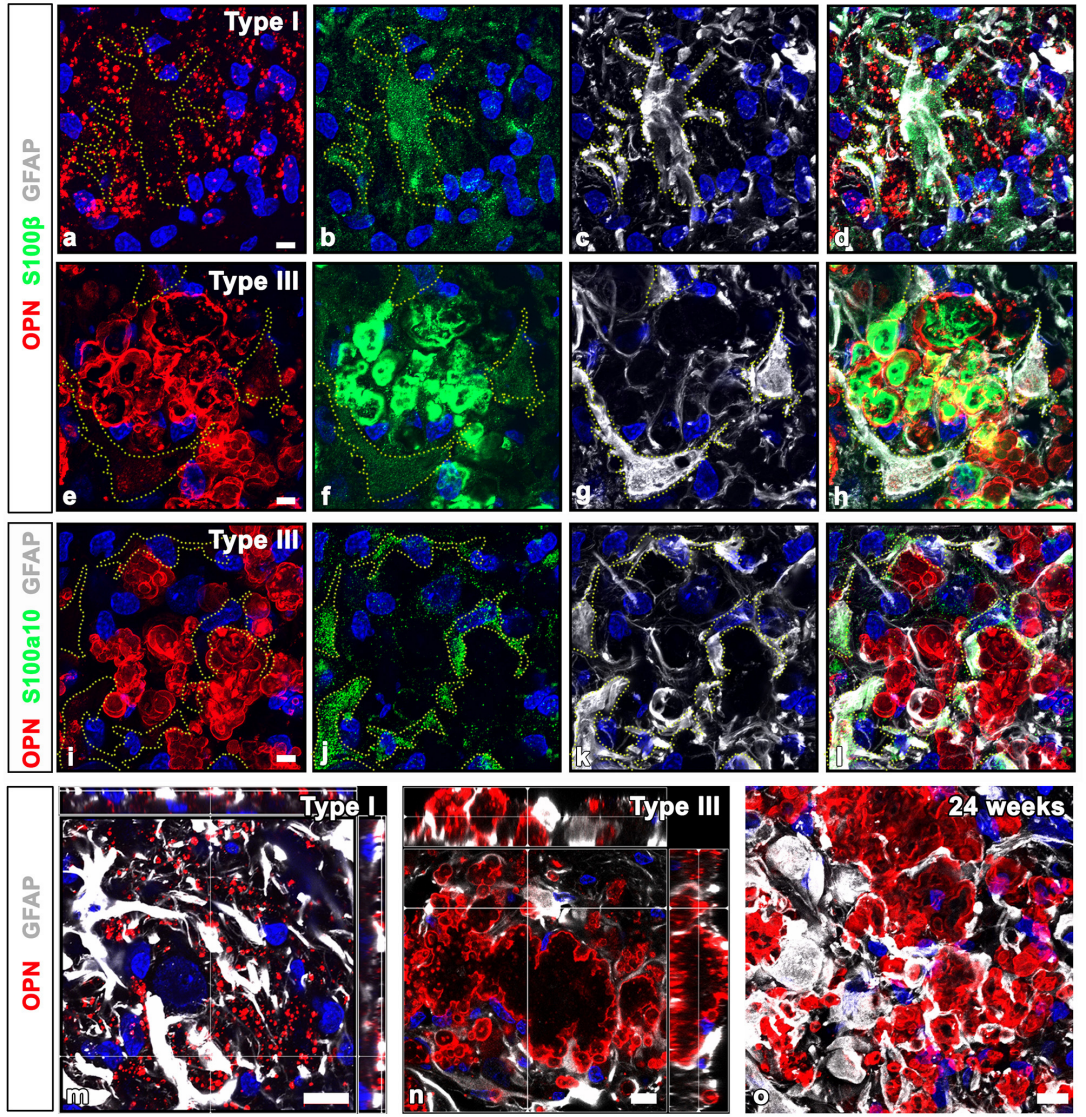
Comparison of GFAP and calcium-binding protein S100β and S100a10 in OPN deposits and astrocytes in the pyramidal cell layer of the ischemic CA1 hippocampus. **(a–d)** Triple labeling for OPN, GFAP, and S100β, showing that type I OPN deposits were closely associated with astrocytes (yellow dotted lines outline cell boundaries), where GFAP and S100β shared largely overlapping distributions, but neither marker was detected within type I OPN deposits. **(e–h)** Triple labeling for OPN, GFAP, and S100β. Type III OPN deposits showed intense S100β expression, but not GFAP. S100β expression within astrocytes (outlined by yellow dotted lines) was much weaker than that of type III OPN deposits. **(i–l)** Triple labeling for OPN, GFAP, and S100a10, showing that S100a10 expression was confined to the GFAP-expressing astrocytes, but not within adjacent type III OPN deposits. **(m, n)** Orthogonal magnified views showing the relationship between OPN deposits and astrocytes in contact with them. **(m)** Type I OPN deposits were mostly scattered in the extracellular space, while some were within astrocytic cytoplasm. **(n)** Type III deposits were mostly located outside astrocytes but were surrounded by them, and only some deposits were located within the astrocytic cytoplasm. **(o)** Type III deposits were still in close contact with astrocytes at 24 weeks after the ischemia. Scale bars = 5 μm for **(a–l)**, 10 μm for **(m-o)**.

Previous studies have reported the presence of S100 calcium-binding proteins in CA structures within the human brain and prostate (Cross et al., 1992; Hoyaux et al., 2000; Fritz et al., 2010). Thus, to ascertain whether CA-like OPN deposits express S100β, a protein that is produced and released mostly by astrocytes, we performed triple labeling for OPN, GFAP, and S100β. Immunoreactivities for GFAP and S100β, both commonly used as astrocytic markers, exhibited different patterns depending on the type of OPN deposits. Type I OPN deposits were closely associated with astrocytes, where GFAP and S100β shared largely overlapping distributions. Conversely, neither marker was detected within these OPN deposits (Figures 5a–d). However, intense S100β expression was observed within type III OPN deposits where the OPN signal was prominent at their peripheral outlines, whereas no GFAP staining was observed within these deposits (Figures 5e–h). Furthermore, S100β expression was observed within astrocytes, although their expression levels were much weaker than those observed within type III OPN deposits (Figures 5e–h). In contrast, astrocytes in contact with type III OPN deposits expressed S100a10, a well-known marker of neuroprotective reactive astrocytes (Liddelow et al., 2017), while these CA-like type III OPN deposits did not exhibit significant S100a10 expression (Figures 5i–l). The spatial relationships between OPN deposits and astrocytes in contact with them were further examined with orthogonal magnified views. Some type I OPN deposits were found within astrocytic cytoplasm, while most were detached and scattered in the extracellular space (Figure 5m). Type II and III deposits showed similar patterns, indicating that only some deposits were located within the astrocytic cytoplasm (Figure 5n). Type III deposits were not cleared away and remained in close contact with astrocytes at 24 weeks after the ischemia (Figure 5o).

**Figure 6:**
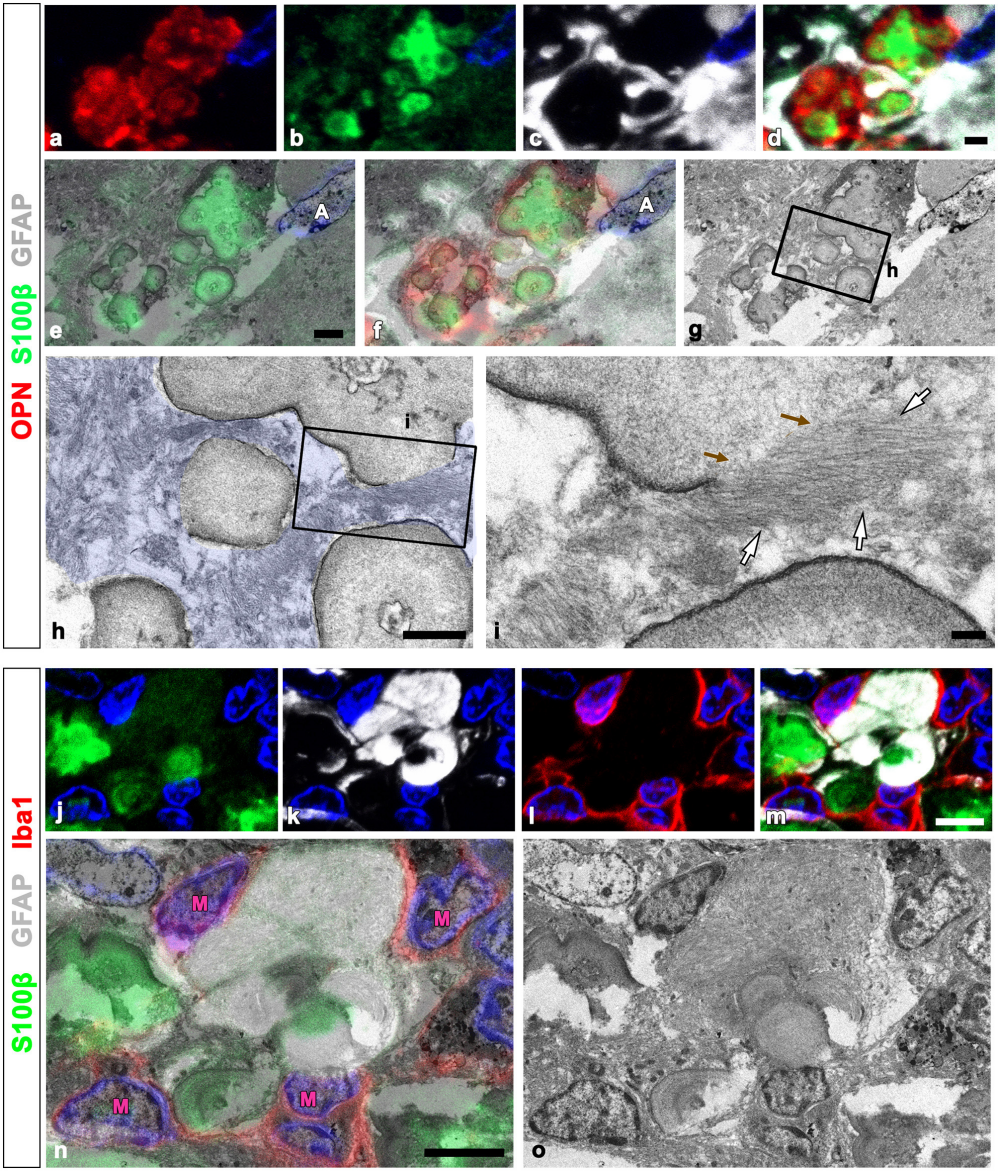
Comparison of precise localization of GFAP and S100β within OPN deposits and adjacent astrocytes using CLEM. **(a–g)** Confocal microscope images of a semi-thin section triple-labeled for OPN, S100β, and GFAP **(a–d)**, the overlay image of S100β expression and the corresponding electron microscopic image **(e)**, the overlay image of the confocal microscope data and the corresponding electron microscopic image **(f)**, and the corresponding electron microscope image **(g)**. **(h, i)** The boxed area in **(g)** was enlarged in **(h)**, and the boxed area in **(h)** was enlarged in **(i)**. Type III OPN deposits with smooth-outlined structures exhibited intense S100β, but not GFAP. Glial fibril bundles [white arrows in **(i)**] appeared attached and fused to type III OPN deposits, but not within preformed deposits. Brown arrows in **(i)** denote the site of astroglial fibrillar attachment to the OPN deposit. **(j–o)** Confocal microscope images of a semi-thin section triple-labeled for S100β, GFAP, and Iba1 **(j–m)**, the overlay image of the confocal microscope data and the corresponding electron microscopic image **(n)**, and the corresponding electron microscope image **(o)**. Concentrically laminated structures, considered OPN deposits, showed intense S100β expression, but lacked glial filaments. These structures were surrounded by astrocytic cytoplasm filled with glial filaments. Microglia (M) frequently observed around these structures showed no obvious morphological changes in activation. Scale bars = 2 μm for **(a–g)**, 1 μm for **(h)**, 0.2 μm for **(i)**, 5 μm for **(j–o)**.

We used the CLEM approach to more clearly compare the precise spatial localization of GFAP and S100β within OPN deposits and adjacent astrocytes. Figures 6a–d depict confocal microscopic images revealing that OPN deposits with intense S100β expression were devoid of GFAP and instead were surrounded by GFAP-positive astrocytes. The overlay of TEM and confocal images revealed that intense S100β, but not GFAP, was detected within type III OPN deposits with smooth-outlined structures (Figures 6e–i). Glial fibril bundles were attached and fused to some OPN deposits; however, the latter comprised uniform materials, with no glial fibril structures observed within them (Figures 6h, i). We performed an additional CLEM experiment with semi-thin sections triple-labeled for GFAP, S100β, and Iba1. As shown in Figures 6j–m, structures with intense S100β expression were surrounded by GFAP-positive astrocytes with relatively weak S100β signals. Additionally, microglia were often observed close to OPN deposits (Figures 6i, m). The overlay of confocal and TEM images revealed concentrically laminated structures that expressed intense S100β expression but were devoid of glial filaments, with these structures being surrounded by astrocytic cytoplasm filled with glial filaments (Figures 6n, o). The microglia frequently observed around these structures displayed no morphological changes characteristic of activation (Figures 6n, o).

**Figure 7:**
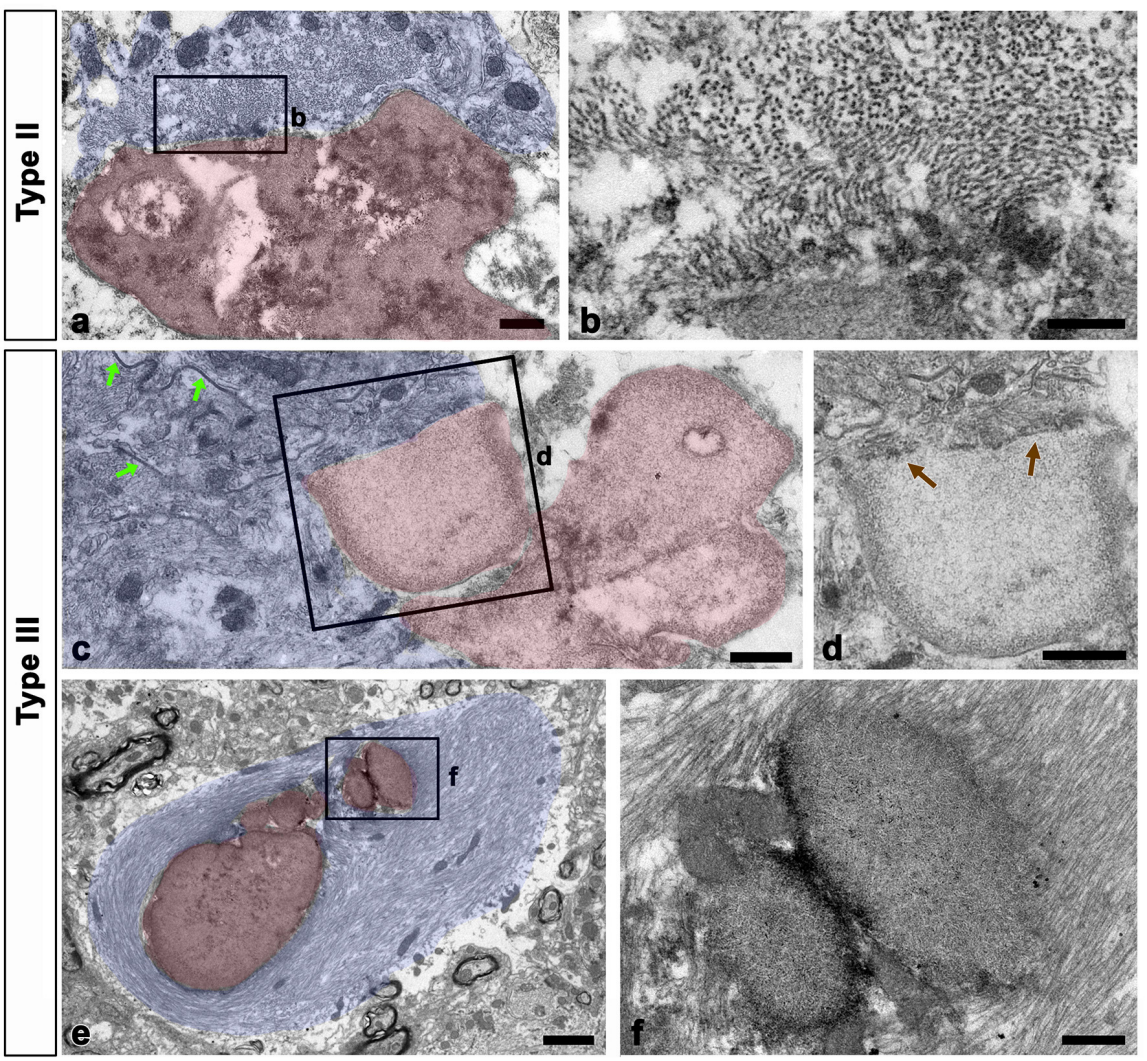
Relationship between glial filaments during OPN deposit formation. **(a–d)** Lower **(a, c)** and higher **(b, d)** conventional electron microscopic images show that astroglia processes (shaded in blue) contained abundant gap junctions [green arrows in **(c)**] and dense bundles of glial filaments attached to the edges of forming types II **(a, b)** and III **(c, d)** OPN deposits (shaded in red). As preformed OPN deposits lacked filaments and cell organelles, a clear boundary [brown arrows in **(d)**] was formed between OPN deposits and astroglia processes. **(e, f)** Lower **(e)** and higher **(f)** conventional electron microscopic images show that type III OPN deposits (shaded in red) are often attached or fused, and they were enwrapped through astroglia processes (shaded in blue). Glial filaments are attached to the outer surface of some deposits. Scale bars = 0.5 μm for **(a)**, 200 nm for **(b)**, 0.5 μm for **(c, d)**, 2 μm for **(e)**, 0.5 μm for **(f)**.

We examined the relationship between glial filaments during OPN deposition using conventional TEM. Bundles of astroglial filaments containing abundant gap junctions appeared attached to the edges of type II and III deposits. However, they were not observed within OPN deposits beyond the attachment site, rendering the boundary clear (Figures 7a–d). Additionally, several small round acellular bodies corresponding to type III OPN deposits, often attached or fused, were enwrapped by astroglial processes filled with glial filaments, with some filaments attached to the outer surface of some deposits (Figures 7e, f).

### 3.5 Ultrastructural relationships between OPN deposits and microglia in the pyramidal cell layer of the CA1 hippocampus after ischemia

**Figure 8:**
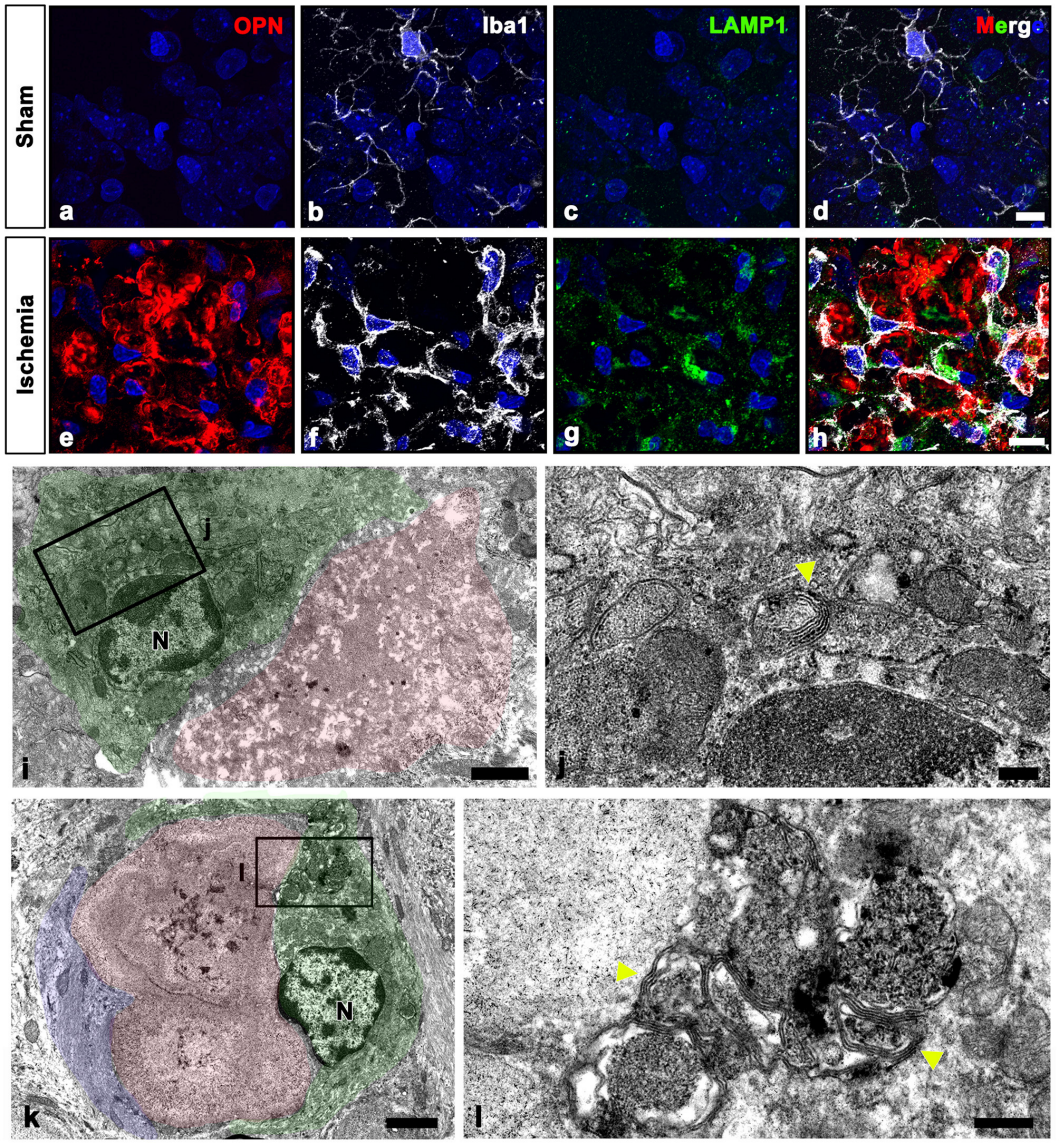
Ultrastructural relationships between OPN deposits and microglia in the pyramidal cell layer of the ischemic CA1 hippocampus. **(a–h)** Triple labeling for OPN, Iba1, and lysosomal-associated membrane protein 1 (LAMP1), a late endosomal/lysosomal marker. Microglia adjacent to OPN deposits in the ischemic hippocampus showed more prominent LAMP1 staining than sham controls. **(i–l)** Lower **(i, k)** and higher **(j, l)** conventional electron microscopic images show that type I **(i, j)** and type III **(k, l)** OPN deposits (shaded in red) were in direct contact with microglia (shaded in green) and astrocytes (shaded in blue). Microglia had dark nuclei with dense and highly clumped heterochromatin (N) and membrane-delineated vuacuoles of autophagic nature (yellow arrowheads) with double or multi-membrane vesicles. Scale bars = 10 μm for **(a–h)**, 2 μm for **(k)**, 1 μm for **(i)**, 0.5 μm for **(l)**, 200 nm for **(j)**.

Our electron microscopy findings demonstrated that microglia were frequently observed around OPN deposits, although these microglia did not exhibit the characteristic morphological changes associated with activation or phagocytosis (Figures 3a, c, 4f, 6o); microglia had a ramified morphology without any OPN deposits within the cytoplasm. To further elucidate the lysosomal activity of nearby microglia in relation to the OPN deposits, we performed triple labeling for OPN, Iba1, and LAMP1, a late endosomal/lysosomal marker. Microglia from sham-operated controls contained only a few LAMP1 -positive lysosomes (Figures 8a–d), while microglia adjacent to OPN deposits in the ischemic hippocampus exhibited prominent LAMP1 staining with a typical punctate pattern. However, OPN deposits were not found within LAMP1-expressing microglia within cell bodies and processes (Figures 8e–h). Using conventional TEM, we observed that astroglia processes surrounded most OPN deposits, but were also in close contact with microglia. Microglia in direct contact with type I (Figures 8i, j) or type III (Figures 8k, l) OPN deposits were characterized by membrane-delineated autophagic vacuoles of different shapes and sizes, mostly exhibiting double- or multi-membrane vesicles with organelles or amorphous electron-dense contents. However, they exhibited well-preserved cell organelles without any phagocytic inclusion of corpora-amylacea like structures, indicating that they were morphologically inactive.

## 4 Discussion

We have previously demonstrated that transient forebrain ischemia induces ongoing neurodegeneration marked by intense Fluoro-Jade B staining in the pyramidal cell layer of the hippocampus at 4 weeks (Riew et al., 2016). Our recent work corroborated this process of delayed neurodegeneration showing that ischemic insults induce three distinct types of OPN deposits based on their morphology and progression in the hippocampus. This process can be regarded as the formation of CA-like structures from degenerative interneurons, co-stained with terminal deoxynucleotidyl transferase dUTP nick end labeling (TUNEL) (Riew et al., 2022). In this study, we suggested that OPN can serve as a surrogate marker and a crucial component of CA and may play an important role in these processes. We revealed that selective calcium deposits initially occur in the mitochondria of degenerated neurons (type I), followed by calcium propagation beyond the mitochondria into the perikarya, forming irregular amorphous structures (type II). Eventually, large calcifying OPN deposits with concentrically laminated structures, typical of CA, were formed (type III). Accordingly, ultrastructural features of degenerated neurons in type I OPN deposits, including electron dense mitochondria and shrunken dark cells with unidentifiable organelles, were demonstrated in the present study. Additionally, we illuminated, for the first time, the role of astrocytes as contributors to CA formation after ischemia.

Using immunoelectron microscopy and CLEM, we observed that reactive astrocytes were in direct proximity to type I OPN deposit-laden degenerating neurons. Additionally, we demonstrated that OPN was synthesized and secreted by reactive astrocytes rather than by activated microglia/macrophages using *in situ* hybridization and pre-embedding immunoelectron microscopy. This finding is further supported by the Golgi-specific localization of OPN in these astrocytes. These results are consistent with our previous study showing that *Opn* mRNA expression was found in microglia up to 3 days after transient global ischemia but shifted to astrocytes in the subacute and chronic phases (Choi et al., 2007). Additionally, OPN is upregulated in reactive astrocytes after events, such as middle cerebral artery occlusion and stab wounds (Zamanian et al., 2012; Sirko et al., 2015). Moreover, a previous study has reported the synthesis and secretion of OPN, the main matrix protein of calcium deposits, by astrocytes rather than by microglia in a mouse model of primary familial brain calcification (Zarb et al., 2021).

Astrocytes were closely associated with all types of OPN, and this pattern became more pronounced from types I to III, with type III deposits almost completely surrounded by astrocytic processes. The spatial relationship between OPN deposits and astrocytes showed that, in the earlier phase, most OPN deposits were located in the extracellular space outside the astrocytes. Gradually, in the later phase, they were surrounded by astrocytes. However, some appeared to be within their cytoplasm, suggesting possible phagocytosis by reactive astrocytes. In particular, the astroglial processes encompassing type III OPN deposits formed multiple layers, displaying upregulated CX43 expression and distinct gap junctions observed through TEM. This finding indicates an active role of reactive astrocytes in post-ischemic CA formation. Astrocytes are interconnected through gap junctions and hemichannels, facilitating intercellular exchange of metabolites and signal transduction (Xing et al., 2019). In postmortem brain samples from patients with Alzheimer's disease (AD) and AD mouse models, reactive astrocytes surrounding plaques exhibit upregulation of gap junction proteins, such as Cx43 and Cx30. This increased expression in reactive astrocytes plays a role in amyloid beta clearance (Nagy et al., 1996; Mei et al., 2010; Kajiwara et al., 2018). Moreover, after cerebral ischemia or hypoxic injury, astrocytic Cx43 regulates extracellular calcium, attenuating cell death and neuroinflammation (Nakase et al., 2003; Li et al., 2015; Liang et al., 2020). In this study, astrocytic processes were closely attached to the types II and III OPN deposits formed during the chronic phase after transient ischemia. These astrocytic processes formed multiple layers, with upregulated CX43 expression and distinct gap junctions observed through TEM, indicating an active role of reactive astrocytes in post-ischemic CA formation.

In some cases, type III OPN deposits in the formation process showed that the smooth-contoured facets were in direct contact with astroglial fibrils. This observation suggests that reactive astrocytes that synthesize and secrete OPN after ischemia might continually interact with degenerating neuronal debris, directly contributing to the formation of CA-like structures. In support of this, an electron microscopy analysis of aged postmortem human hippocampi reported that the CA is encompassed by processes that are rich in intermediate glial filaments (Augé et al., 2019). Furthermore, studies analyzing periodic-acid schiff (PAS) granules in mice, which are considered analogous to human CA, have demonstrated that the proteins constituting these granules are expressed in the fine processes of reactive astrocytes. This finding suggests that CA is more likely to be formed by astrocytes rather than microglia (Augé et al., 2018b; Wander et al., 2020, 2022). Astroglial fibrils that were attached to the forming surface of the OPN deposits were not detected in the preformed deposits beyond the site of the fibrillar attachment. This finding was further supported by the absence of GFAP expression within the OPN deposits enclosed by GFAP-positive astroglial processes. These data suggest that astroglial fibrils may directly intervene in the formation of CA-like structures, but that the fibril may not act as a component of CA-like structures or may have altered structural features within them. Therefore, further studies are required to determine the precise role of astroglial fibrils in CA formation.

Our previous research has highlighted that post-ischemic OPN deposits exhibit calcifying profiles (Park et al., 2012; Riew et al., 2017, 2022). Studies using animal models of primary familial brain calcification and patient brain samples have reported that astrocytes and microglia surround calcified nodules (Keller et al., 2013; Nahar et al., 2019; Zarb et al., 2021; Maheshwari et al., 2023). However, OPN, the key matrix protein of calcified nodules, is expressed by astrocytes rather than microglia (Zarb et al., 2021), which is consistent with our present findings confirming the expression of OPN mRNA and protein localized to the Golgi complex. Another study on primary brain calcification showed that astrocytes completely encompassed fully-formed calcium nodules and contained intracellular deposits (Jensen et al., 2018). Interestingly, the CLEM approach revealed that astrocytic cytoplasm, containing dense glial bundles and cell organelles, was in close proximity to calcified mitochondria (type I deposits) within degenerating neurons (see Figures 4h–j). Therefore, it is assumed that astrocytes are closely associated with degenerative cells and secrete OPN to be involved in their calcification processes, which begin with intramitochondrial calcification and subsequently propagate calcification throughout the entire cell. Considering these previous findings and our observations, astrocytes may synthesize and secrete OPN, a matrix protein of calcium deposits, and actively participate in the formation of calcium deposits.

The active formation of calcium deposits and CA-like structures by astrocytes is further supported by the expression of an astrocytic cytoplasmic protein, S100β, within the calcium deposits. S100β is a calcium-binding protein participating in dynamic cellular physiology (Sorci et al., 2010), and S100 proteins are well-known components of CA in aged human brain (Hoyaux et al., 2000; Augé et al., 2018a). Additionally, S100 proteins exhibit self-aggregation and amyloidogenic potential under specific conditions and are implicated in the dystrophic calcification of the CA in peripheral organs (Fritz et al., 2010). S100β protein modulates amyloid beta plaque formation in AD (Cristóvão et al., 2018). Astrocytes were found in close contact with all types of OPN deposits; however, the intensity of S100β expression within the fully-formed type III deposits, calcified CA, was much stronger compared to astrocytes. On the other hand, S100a10, a well-known marker of neuroprotective reactive astrocytes (Liddelow et al., 2017), was highly expressed in astrocytes contacting type III deposits, but these CA-like deposits were devoid of S100a10. This suggests that S100β, secreted by astrocytes but not S100a10, could accumulate along with OPN and calcium, contributing to CA formation. However, further research is required to understand the specific interactions between these molecules and the temporal relationship between molecular secretion and CA formation.

Another interesting observation in this study is that although microglia surrounding the OPN deposits showed upregulation of lysosomal marker LAMP1 and contained numerous autophagic vacuoles, they maintained contact without direct engulfment of the deposits and had morphologically inactive structures. Similarly, microglia enveloping calcium nodules in the animal model of primary familial brain calcification displayed characteristics of activated microglia, showing upregulation of genes, such as Cd68 and Clec7a, and enrichment of inflammatory pathway genes. However, direct phagocytosis of calcification has not been reported (Zarb et al., 2021). Microglia observed in this primary familial brain calcification model were of a distinct morphological phenotype known as “dark microglia,” characterized by electron-dense cytoplasm and associated with neurodegeneration (Maheshwari et al., 2023). This suggests that microglia differentially regulate calcification based on context but are not the main cells involved in CA formation or phagocytosis.

In conclusion, our results indicate that astrocytes may be involved in the entire process, from the initial formation of type I OPN deposits through the evolution of types II and III deposits to the reorganization of CA-like structures. Summarily, astrocytes (1) synthesize and secrete OPN, a surrogate marker and principal composition molecule of CA and calcium deposits, (2) actively participate in the formation of calcium deposits, and (3) secrete S100β, which is a component of CA and can aggregate under interaction with calcium. Collectively, our study is the first to demonstrate that astrocytes are the primary cells responsible for post-ischemic CA formation.

## Data availability statement

The raw data supporting the conclusions of this article will be made available by the authors, without undue reservation.

## Ethics statement

The animal study was approved by Institutional Animal Care and Use Committee (IACUC) at the College of Medicine of The Catholic University of Korea. The study was conducted in accordance with the local legislation and institutional requirements.

## Author contributions

T-RR: Conceptualization, Funding acquisition, Investigation, Methodology, Visualization, Writing – original draft, Writing – review & editing. J-WH: Investigation, Methodology, Validation, Writing – review & editing. XJ: Investigation, Methodology, Validation, Writing – review & editing. HK: Methodology, Validation, Visualization, Writing – review & editing. SJ: Methodology, Visualization, Writing – review & editing. M-YL: Conceptualization, Funding acquisition, Project administration, Supervision, Writing – original draft, Writing – review & editing.
